# Systemic Cystic Angiomatosis Mimicking Metastatic Cancer: A Case Report and Review of the Literature

**DOI:** 10.1155/2017/5032630

**Published:** 2017-09-11

**Authors:** Vivek Kumar, Trishala Meghal, Yanyu Helen Sun, Yiwu Huang

**Affiliations:** ^1^Department of Internal Medicine, Maimonides Medical Center, Brooklyn, NY, USA; ^2^Department of Hematology and Oncology, Maimonides Cancer Center, Brooklyn, NY, USA; ^3^Department of Pathology, Maimonides Medical Center, Brooklyn, NY, USA

## Abstract

Systemic cystic angiomatosis is a rare benign disorder due to the maldeveloped vascular and lymphatic system with less than 50 cases reported in literature so far. We report here a case of systemic cystic angiomatosis (SCA) with multisystem involvement affecting the neck, thyroid, thoracic cavity, and skeletal system. The patient initially presented in her 4th decade of life with isolated lymphangioma in the neck requiring surgery. However, she experienced full-blown manifestations of SCA in her 6th decade which closely mimicked metastatic cancer. The diagnosis of SCA could only be established after multiple biopsies. The radiological and histological features of SCA with its course over 31 years in this patient have been described.

## 1. Introduction

Systemic cystic angiomatosis (SCA) is a rare disorder characterized by multicystic vascular involvement of the skeletal system but occasionally it can also affect other organ systems [1]. Its pathology most likely represents a maldeveloped vascular and/or lymphatic system [2]. Most of the patients present around puberty [3]. Herein we describe a rare case of SCA with late presentation in whom the disease first manifested after 4th decade with recurrent lymphangiomas but the full-blown disease presented only after 6th decade mimicking a metastatic cancer.

## 2. Case Presentation

A 67-year-old woman in otherwise good health presented to the emergency room (ER) in 2010 with severe pain over back and rib cages bilaterally, associated with shortness of breath for one week. On review of systems, she also reported fatigue and dry cough. She denied any fever, chills, nausea, vomiting, weight loss, and loss of appetite. Her past medical history was significant for neck and thyroid gland surgeries in 1986 and 1998, respectively, for masses which were diagnosed postoperatively as lymphangiomas on both occasions. Family history was unremarkable.

**Figure 1:**
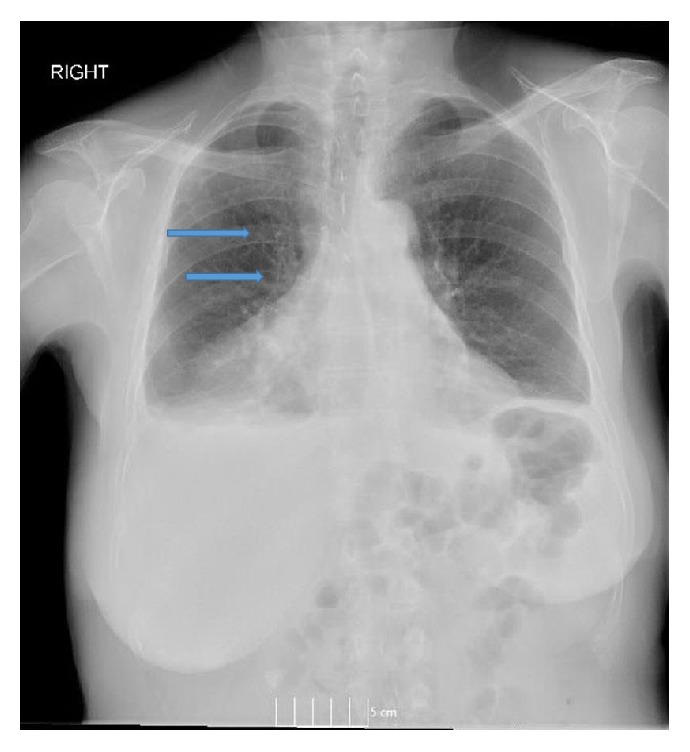
Chest X-ray (Posteroanterior view) showing bilateral pleural effusions with an expansile lucent lesions with septations in the right fourth and fifth ribs (arrow).

**Figure 2:**
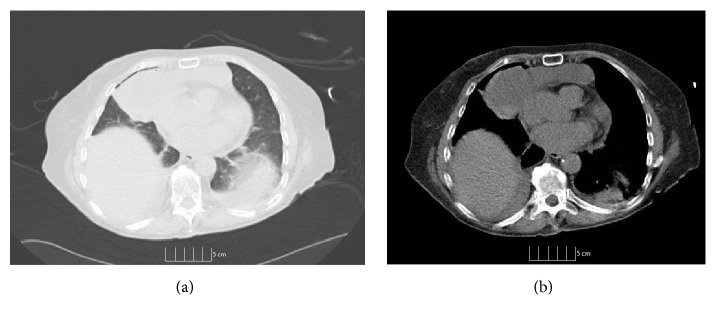
Cystic lesion in the mediastinum on (a) noncontrast CT lung window and (b) noncontrast CT of the chest.

**Figure 3:**
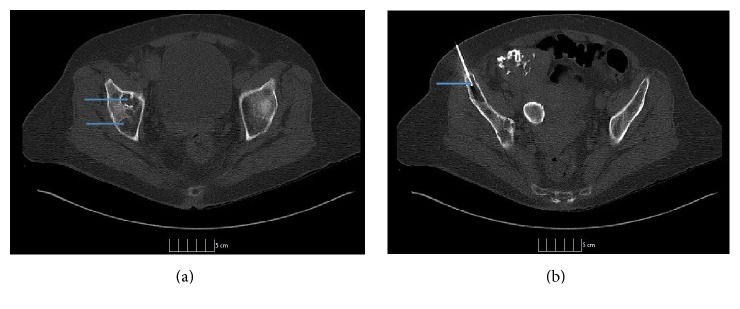
CT imaging of the pelvis showing cystic osteolytic (exceptional) bony lesion with a sclerotic rim within the (a) right acetabulum and (b) right iliac crest with FNAC needle.

On physical examination, she was not in distress and could speak in full sentences. She had no cyanosis, clubbing, raised jugular venous pressure (JVP), pedal edema, or lymphadenopathy. The chest wall and spine were nontender on palpation. The respiratory system examination revealed decreased breath sounds over the right side of chest and on auscultation; crackles were heard over the right chest. The examination of abdominal, cardiovascular, and neurological systems was nonrevealing. The blood investigations revealed mild leukocytosis (11,800 per microliter) with neutrophilia (82%), mild thrombocytosis (491,000 per microliter), hypoproteinemia (6.2 gm per deciliter), and hypoalbuminemia (3.2 gm per deciliter). Lactate dehydrogenase (LDH) (109 units per liter) and alkaline phosphatase (ALP) were normal (32 International Units per liter). The chest X-ray showed bilateral pleural effusions with atelectasis. It also showed expansile lucent lesions with septations in the right fourth and fifth ribs (Figure 1). The computed tomography (CT) scan revealed a large loculated pleural effusion with loculated fluid in the anterior mediastinum, a 3 cm mass in the right cardiophrenic angle, a 3 mm right lower lobe lung nodule, and two hypoenhancing lesions in the right hepatic lobe which were too small to be characterized (Figures 2(a) and 2(b)). It also showed a lytic bubbly expansile lesion at the right transverse process of T3 and a smaller lytic lesion identified at the posterolateral fourth rib, with multiple scattered lytic lesions demonstrated within the thoracic vertebral bodies and posterior elements. Due to the high suspicion of malignancy, patient underwent CT-guided biopsy of the mediastinal mass with aspiration of serosanguinous fluid. The histopathology showed connective tissue with reactive fibroblasts suggestive of granuloma, but there was no evidence of malignant cells. Immunohistochemistry was negative for AE1/AE3, CD3, CD20, PAX-5, CD117, CD138, kappa, and lambda, which ruled out any solid or hematological malignancy. Additional workup including serum electrophoresis and flow cytometry was also normal. The X-ray of the femur revealed multiple lesions involving the right femur and right iliac bone with endosteal scalloping, lytic and moth eaten lesions suspicious for metastatic disease (Figure 3(a)). The bone scan showed absence of generalized uptake; instead it showed an ill-defined patchy focus of mildly increased uptake at the right mid femur. A second biopsy was taken from the iliac bone by interventional radiologist (IR) which only showed atypical cells and was nondiagnostic (Figure 3(b)). The diagnosis could not be established despite two biopsies. Magnetic Resonance Imaging (MRI) of the pelvis also reported multiple intraosseous lesions at the sacrum, ilium, pubic rami, and proximal femur. It was suspicious for extensive metastatic disease of the spine, pelvis, and proximal femur. After an interdepartmental discussion it was decided to obtain an open surgical biopsy from the lesions on the right iliac crest and proximal femur. It showed rare clusters of histiocytes but diagnosis could not be established. The tissues were sent to a higher center for the second opinion. It revealed multiple dilated vascular channels with flat endothelial like cells in the bone biopsies and mediastinal lesion with predominantly lymphangiomatous process (Figures 5(a) and 5(b)). On immunohistochemistry (IHC), these cells were focally positive for CD31 and D2-40 which suggested their origin from lymphovascular endothelium (Figure 5(c)). Thus the diagnosis of lymphangiomatous variant of systemic cystic angiomatosis (SCA) was established.

**Figure 4:**
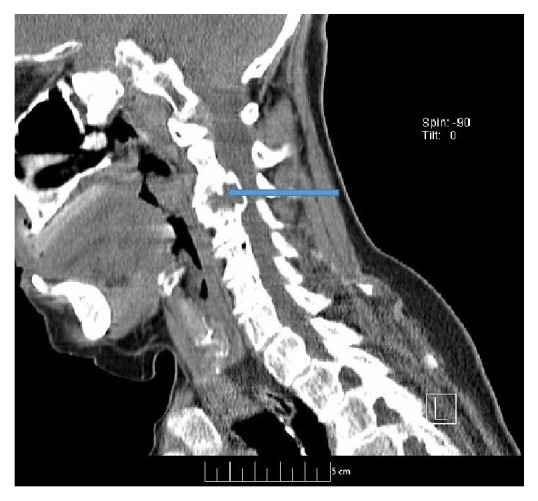
Sagittal CT of cervical spine image showing expansile lesion at C3 with preserved surrounding cortex.

**Figure 5:**
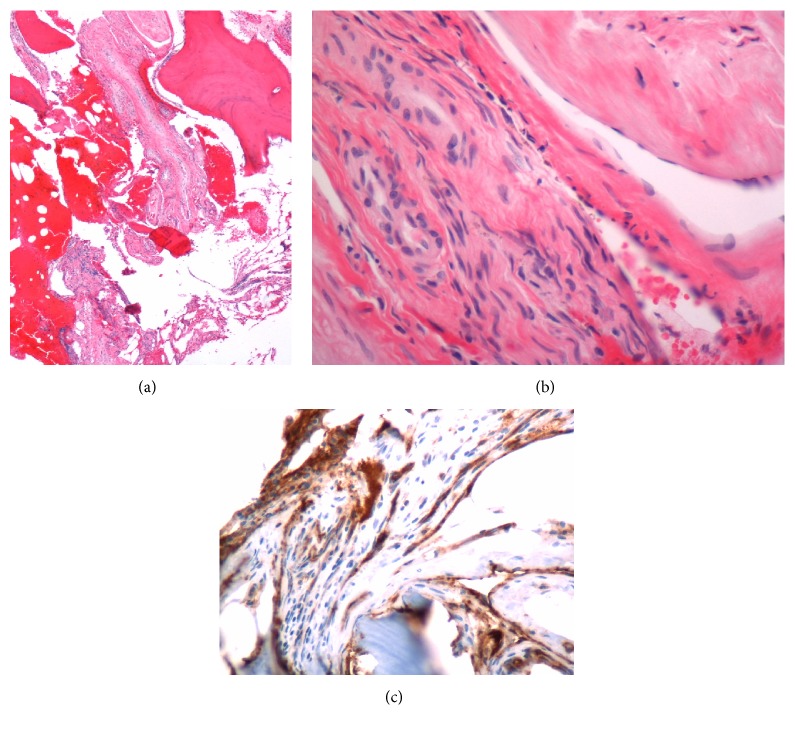
(a) Histological section showing multiple dilated vascular channels of irregular sizes with flat endothelial cells; (b) anterior mediastinal biopsy showing findings suggestive of a cyst wall; (c) immunohistochemistry stain for D2-40 highlighting lymphovascular endothelium.

The patient was started on parenteral zoledronic acid 4 mg intravenously every 4 weeks due to extensive skeletal involvement. In 2013, she complained of lower back ache. An MRI was obtained which revealed mild degenerative changes with anterior vertebral spurring in the lower thoracic levels and at L2-L3 levels. There was intervertebral disk space narrowing at T11-T12. The bone marrow signal demonstrated a rounded area of T2 hyperintensity and T1 hypointensity in the lumbar vertebra, posterior elements, iliac wings, and sacrum consistent with systemic cystic angiomatosis. A CT scan of cervical spine was also obtained which showed multiple expansile lesions throughout the cervical and thoracic spine which were most pronounced at C3, producing mild right paratracheal canal stenosis and severe right neuroforaminal stenosis at C3 and C4 (Figure 4). She responded to physical therapy and analgesics. In 2016, she presented to ER again with complain of severe neck pain. The signs and symptoms did not suggest neurological involvement. MRI of cervical spine showed compression fracture of C3. She was evaluated by neurosurgery who did not recommend any surgical intervention at this point. Patient was managed with cervical spine stabilization and pain medications. She was discharged after a short hospital stay. CT scan of chest, abdomen, and pelvis in 2012, 2014, and 2016 showed stable disease without any progression. The frequency of zoledronic acid was changed to every 3 months in 2016. Patient has recovered completely from vertebral compression fracture now and has no pain or neurological deficits and is under regular follow-up.

## 3. Discussion

This case highlights a very rare disease which could only be diagnosed after open surgical biopsy, 25 years after its initial presentation. Interestingly, SCA is a benign disease but follows a progressive course and can lead to catastrophic outcomes due to visceral involvement or cervical spine fractures leading to neurological complications and deaths [4]. Due to the late presentation in our patient, it closely mimicked a widely metastatic disease due to multiorgan involvement, especially bony lesions. Over 25 years, this patient was operated on twice for anticipated malignancies but histopathological examination of postsurgical specimens established the diagnosis of benign lymphangiomas on both occasions. The diagnosis could only be established after multiple biopsies and consultation with specialized center.

Najm et al. recently reviewed the published literature and, including their 4 cases, only 48 patients have been reported in the 36 case reports so far [3]. Mean age of presentation was 22 years; however bimodal distribution of age has been suggested due to late presentation in some cases [3, 5–7]. Men seem to be affected more frequently than women [4]. The etiology is unknown but most like results from maldeveloped vascular and/or lymphatic systems [2]. The presentation of SCA is highly variable. Majority of the cases presented with the bony cysts affecting most commonly the femur and pelvic bones [8, 9]. Spinal involvement is seen in up to 50% patients [10]. Rarely, it can also affect the small bones of hands [11]. Visceral involvement is seen in up to 35% of reported cases. Spleen is the most common organ which is affected in one-fourth of cases [3]. Other sites include skin and soft tissues, thymus, liver, kidneys, mediastinum, and mesentery. Lungs and pleural involvement is uncommon [11]. To the best of our knowledge, we are reporting the index case of thyroid involvement in SCA. The complications include bony pains, pathological fractures, deformities, splenic rupture, chylothorax, and spinal cord compression [3, 4, 12]. Alkaline phosphatase has been reported as elevated in few reports due to extensive lytic osseous lesions; however it has been reported normal in many other reports including in this patient [13].

Radiologically, the characteristic lesions of SCA appear well-defined intramedullary cysts with preservation of cortical bone surrounded by a sclerotic rim without any periosteal reaction [1]. The lytic lesions similar to this patient have not been described frequently in SCA and clinch diagnosis in favor of Gorham-Stout disease (GSD), also known as disappearing or vanishing bone disease [14]. However the histological and MRI findings in this case were classical for SCA. The bone scan only showed mild abnormalities while MRI showed multiple intraosseous lesions at the hip bone and lucent expansile lesions were present in the fourth and fifth ribs. This discrepancy is most likely due to the inhibition of osteoblastic differentiation by the aggressive lymphangiomatosis which also explains the lack of a sclerotic rim around the cysts in this patient. The sclerosing form has been reported in elderly patients in the previous cases and osteolysis was more common in the younger patients [3]. However this patient presented with lytic bony lesions in her 6th decade of life. This cannot be explained with the current concept of its pathology where osteolysis is considered to be the initial step which usually starts in early age followed by sclerosis later which is common in advanced age [15]. This suggests that our understanding of disease spectrum and biology is limited owing to its rarity. Due to involvement of multiple bones, it mimics bone metastasis, lymphoma, multiple myeloma, Paget's disease, and histiocytosis. Establishing a diagnosis is extremely difficult due to its rarity and lack of awareness about this entity.

The pathogenesis of SCA is poorly understood. The abnormal presence of lymphatic vessels in the bones and their uncontrolled proliferation lead to formation of cysts. However this is considered to be a developmental anomaly instead of a tumor. This proliferation may be stimulated by several prolymphangiogenic factors of VEGF and angiopoietin factors. The SCA appears to have genetic bases due to the clustering of cases in some families. Autosomal dominance pattern of inheritance has been suggested but requires confirmation [16].

There is no specific treatment of this disease. However the course is variable including progressive in some cases like the present case and stable in some [4]. Spontaneous regression leading to cure has also been reported [17]. Bisphosphonates decrease osteolysis and increase mineralization by inhibiting the osteoclast activity in high turn-over states [18]. They are also known to exert antiangiogenic effect through inhibition of endothelial cells proliferation and by triggering their apoptosis. Mainly zoledronic acid controls the expression of angiogenic cytokines like vascular endothelial growth factor (VEGF), basic fibroblast growth factor (bFGF), and platelet derived growth factors (PDGF), thereby modulating migration and adhesion of endothelial cells. This possibly stabilizes the bony lesions and causes fewer skeletal related events [19]. However actual benefit from any kind of treatment is not clear. The dose and agent of choice among bisphosphonates for the treatment of patients with SCA is not clear. We used zoledronic acid due to its additional effect on angiogenic modulation. The usual dose of zoledronic acid for the treatment of patients with hypercalcemia of malignancy is 4 mg as single dose which may be repeated after 7 days if indicated while its dose in multiple myeloma or osseous metastases from solid tumors is 4 mg every 3-4 weeks [20]. The use of zoledronic has been reported previously in the patients with SCA [3]. Najm et al. reported using zoledronic acid 4 mg once every 6 months for two years followed by every year. They reported occurrence of new lesions at 2 years but followed by no new lesions for next 4 years [3]. Marcucci et al. also reported use of pamidronate (30 mg IV every month) for two years followed by zoledronic acid (5 mg once a year) in a 20-year-old patient with vertebral column involvement [19]. They reported slight improvement in bone mineral density after pamidronate treatment for 2 years and no change in the cystic lesions. After switching over to zoledronic acid, they reported marked improvement in the BMD with stabilization of cystic lesions at 5 and 7 years from start of bisphosphonate treatment. The monthly use of zoledronic acid has been reported in patients with GSD which is more severe form of angiomatosis [21, 22]. Due to extensive skeletal involvement with lytic lesions similar to GSD and involvement of cervical spine, we decided to treat her with monthly doses of zoledronic acid. The patient is tolerating the treatment well with normal renal function and her disease is stable for seven years now.

## 4. Conclusion

CSA is a heterogeneous disease with unpredictable course. Despite being a benign disease, the disease course may be complicated by occurrence of life-threatening complications. The diagnosis is extremely difficult and often mimics malignancy especially in old age. High index of suspicion is required to establish the accurate diagnosis.
